# Co-expression of putative stemness and epithelial-to-mesenchymal transition markers on single circulating tumour cells from patients with early and metastatic breast cancer

**DOI:** 10.1186/1471-2407-14-651

**Published:** 2014-09-03

**Authors:** Maria A Papadaki, Galatea Kallergi, Zafeiris Zafeiriou, Lefteris Manouras, Panayiotis A Theodoropoulos, Dimitris Mavroudis, Vassilis Georgoulias, Sofia Agelaki

**Affiliations:** Laboratory of Tumor Cell Biology, School of Medicine, University of Crete, GR-71110 Heraklion, Crete Greece; Department of Medical Oncology, University Hospital of Heraklion, GR-71110 Heraklion, Crete Greece; Laboratory of Biochemistry, School of Medicine, University of Crete, GR-71110 Heraklion, Crete Greece

## Abstract

**Background:**

The detection of circulating tumor cells (CTCs) in peripheral blood (PB) of patients with breast cancer predicts poor clinical outcome. Cancer cells with stemness and epithelial-to-mesenchymal transition (EMT) features display enhanced malignant and metastatic potential. A new methodology was developed in order to investigate the co-expression of a stemness and an EMT marker (ALDH1 and TWIST, respectively) on single CTCs of patients with early and metastatic breast cancer.

**Methods:**

Triple immunofluorescence using anti-pancytokeratin (A45-B/B3), anti-ALDH1 and anti-TWIST antibodies was performed in cytospins prepared from hepatocellular carcinoma HepG2 cells and SKBR-3, MCF-7 and MDA.MB.231 breast cancer cell lines. Evaluation of ALDH1 expression levels (high, low or absent) and TWIST subcellular localization (nuclear, cytoplasmic or absent) was performed using the ARIOL system. Cytospins prepared from peripheral blood of patients with early (n = 80) and metastatic (n = 50) breast cancer were analyzed for CTC detection (based on pan-cytokeratin expression and cytomorphological criteria) and characterized according to ALDH1 and TWIST.

**Results:**

CTCs were detected in 13 (16%) and 25 (50%) patients with early and metastatic disease, respectively. High ALDH1 expression (ALDH1^high^) and nuclear TWIST localization (TWIST^nuc^) on CTCs was confirmed in more patients with metastatic than early breast cancer (80% vs. 30.8%, respectively; p = 0.009). In early disease, ALDH1^low/neg^ CTCs (p = 0.006) and TWIST^cyt/neg^ CTCs (p = 0.040) were mainly observed. Regarding co-expression of these markers, ALDH1^high^/TWIST^nuc^ CTCs were more frequently evident in the metastatic setting (76% vs. 15.4% of patients, p = 0.001; 61.5% vs. 12.9% of total CTCs), whereas in early disease ALDH1^low/neg^/TWIST^cyt/neg^ CTCs were mainly detected (61.5% vs. 20% of patients, p = 0.078; 41.9% vs. 7.7% of total CTCs).

**Conclusions:**

A new assay is provided for the evaluation of ALDH1 and TWIST co-expression at the single CTC-level in patients with breast cancer. A differential expression pattern for these markers was observed both in early and metastatic disease. CTCs expressing high ALDH1, along with nuclear TWIST were more frequently detected in patients with metastatic breast cancer, suggesting that these cells may prevail during disease progression.

**Electronic supplementary material:**

The online version of this article (doi:10.1186/1471-2407-14-651) contains supplementary material, which is available to authorized users.

## Background

Circulating tumor cells (CTCs) have been identified in peripheral blood (PB) of patients with breast cancer and their presence has been associated with poor disease outcome [1–4]. It has been suggested that CTCs are extremely heterogeneous and that they include the population of cells giving rise to overt metastases [5]. Therefore further characterization of CTCs at the single cell level would be of utmost importance in order to understand their individual biologic role.

Several studies in many tumor types, including breast cancer, reported that there is a subset of cells with stemness properties, named cancer stem cells (CSCs). These cells are proposed to display enhanced malignant and metastatic potential [6–8]. Tumor cells with increased activity of the detoxifying enzyme aldehyde dehydrogenase (ALDH) are considered as putative breast CSCs, due to their self-renewal capacity as shown by serial passages in Nonobese Diabetic/Severe Combined Immunodeficiency (NOD/SCID) mice and their ability to regenerate the cellular heterogeneity of the initial tumor [9]. Ginestier et al., showed a correlation between ALDH activity and ALDH1 expression in breast cancer cells [10]. Moreover, the expression of ALDH1 in primary tumors has been associated with poor prognosis in patients with breast cancer [10–12]. We, among others, have recently reported that CTCs expressing ALDH1 are detectable in patients with metastatic breast cancer, suggesting that this “stemness phenotype” could be related to metastases formation [13, 14].

There is growing evidence suggesting that both tumor growth and metastatic dissemination take place through a phenotypic modulation known as epithelial-to-mesenchymal transition (EMT), a process by which tumor cells lose their epithelial characteristics and acquire a mesenchymal phenotype [15, 16]. TWIST, a basic helix-loop-helix transcription factor has been proposed among others as a putative biomarker for EMT [17, 18]. A positive association between the expression of TWIST in primary tumors and the risk for recurrence and poor survival has been shown in breast cancer [19–21]. Moreover, we have recently reported that TWIST expressing CTCs are frequently observed in patients with breast cancer [22, 23], suggesting that cancer cells might undergo EMT during vessel invasion, circulation and migration to metastatic sites.

Recent studies have shown a direct link between CSCs and EMT in breast cancer, suggesting that EMT generates cancer cells with stem cell-like traits [24–26]. Co-expression of stem cell and EMT markers at the mRNA expression level has been shown on CTCs of breast cancer patients [27, 28]; however, this has not been demonstrated on single CTCs as yet. Taking into account the considerable heterogeneity of CTCs, the presence of both stemness and EMT characteristics on individual CTCs could distinguish a population of cells with enhanced metastatic potential.

In the present study we developed a new methodology using the ARIOL system, in order to evaluate the protein expression pattern of a putative stemness (ALDH1) and an EMT (TWIST) marker on CTCs of early and metastatic breast cancer patients. We aimed to investigate the co-expression of these markers at the single CTC-level and to evaluate the incidence of distinct CTC subpopulations in early and metastatic disease.

## Methods

### Patient samples

Peripheral blood (10 ml) was obtained from patients with early (n = 80) and metastatic (n = 50) breast cancer, before the initiation of adjuvant and first-line chemotherapy, respectively. In order to avoid contamination with epithelial cells derived from the skin, blood was obtained at the middle of vein ^P^puncture, after the first 5 ml were discarded. Peripheral blood mononuclear cells (PBMCs) cytospins were prepared and stored until use. In the current study, prospectively collected cytospins were analyzed. Peripheral blood was also obtained from healthy blood donors (n = 20). All patients and healthy volunteers gave their written informed consent to participate in the study, which has been approved by the Ethics and Scientific Committees of the University General Hospital of Heraklion, Crete, Greece.

### Cytospin preparation

PBMCs were isolated by Ficoll-Hypaque density gradient (d = 1,077 gr/mol) centrifugation at 1.800 rpm for 30 min. PBMCs were washed two times with phosphate-buffered saline (PBS) and centrifuged at 1.600 rpm for 10 min. Aliquots of 250.000 cells were cyto-centrifuged at 2.000 rpm for 2 min on glass slides. Air-dried cytospins were stored at −80°C.

### Cell cultures

All cell lines were obtained from American Type Culture Collection (ATCC). The HepG2 (human liver hepatocellular carcinoma), MCF-7 and MDA.MB.231 cells were cultured in high glucose GlutaMAX^(^™^)^ Dulbecco’s Modified Eagle Medium (DMEM) (GIBCO-BRL Co, MD, USA), supplemented with 10% fetal bovine serum (FBS) (GIBCO-BRL) and 1% penicillin/streptomycin (GIBCO-BRL). MCF-7 cell culture medium was additionally supplemented with 0.28% insulin. SKBR-3 cells were cultured in high glucose GlutaMAX^(^™^)^ McCoys5A medium (GIBCO-BRL) supplemented with 10% FBS and 1% penicillin/streptomycin. Cells were maintained in a humidified atmosphere of 5% CO_2_- 95% air at 37°C. Subcultivation of all cell lines was performed using 0.25% trypsin and 5 mM ethylenediaminetetraacetic acid (EDTA) (GIBCO-BRL).

### Immunofluorescence assay

PBMCs’ cytospin preparations were triple-stained with pan-cytokeratin, ALDH1 and TWIST. Cytokeratin-positive cells were detected using the A45-B/B3 anti-mouse antibody (recognizing the CK8, CK18 and CK19; Micromet, Munich, Germany). PBMCs’ cytospins were also double-stained with pan-cytokeratin and CD45 (common leukocyte antigen), in order to exclude possible ectopic expression of cytokeratins in hematopoietic cells, as previously described [29, 30]. As proposed by Meng et al. [31], the cytomorphological criteria of high nuclear to cytoplasmic ratio and size larger than white blood cells, were also employed in order to characterize a cytokeratin-positive cell as a CTC.

PBMCs’ cytospin preparations were fixed with 3% (v/v) paraformaldehyde (PFA) in PBS for 30 min and permeabilized with 0.5% Triton X-100 in PBS for 10 min at room temperature (RT). After an overnight blocking with PBS supplemented with 1% Bovine Serum A (BSA) at 4P^oP^C, cells were double-stained for pan-cytokeratin/CD45 or triple-stained for pan-cytokeratin/ALDH1/TWIST. The incubation time for all primary and secondary antibodies was 1 h and 45 min, respectively. Zenon technology (FITC-conjugated IGg1 antibody) (Molecular Probes, Invitrogen) was used for the detection of pan-cytokeratin (A45-B/B3 anti-mouse antibody). CD45 was detected using an anti-rabbit antibody (Santa Cruz, CA, USA) labelled with Alexa 555 (Molecular Probes, Invitrogen, Carlsbad, CA, USA); ALDH1 was detected using an anti-mouse antibody (Abcam, Cambridge, UK) labelled with Alexa 555 (Molecular Probes); TWIST was detected using an anti-rabbit antibody (Abcam) labelled with Alexa 633 (Molecular Probes). Cells were post-fixed with 3% (v/v) PFA in PBS for 15 min at RT. Dapi-antifade reagent (Invitrogen) was finally added to each sample for cell nuclear staining.

A total of 500.000 PBMCs per patient were analyzed using the ARIOL system CTCs software (Genetix, UK) as previously described [22]. Results are referred to patients with detectable CTCs only and are expressed as number of CTCs/500.000 PBMCs.

### Evaluation of sensitivity and specificity of CTC detection

The sensitivity of CTC detection using the current methodology was evaluated by two separate approaches; MCF-7, SKBR-3 and MDA.MB.231 breast cancer cells were spiked into separate aliquots of 10 ml peripheral blood obtained from ten healthy female blood donors, at a concentration of 1, 10 and 100 cells per ml. Furthermore, MCF-7 cells were spiked into separate aliquots of 10*10^6^ PBMCs from healthy volunteers, at a concentration of 1, 10 and 100 cells per 1*10^6^ PBMCs. All samples were processed as previously described for patients’ samples.

To determine the specificity of CTC detection, peripheral blood was obtained from ten healthy donors and samples were also processed as described above. Furthermore, cytospins of HepG2 cells spiked into healthy donors’ PBMCs (100/250.000 PBMCs) were used as positive and negative controls in order to evaluate the specificity of all antibodies. Negative controls were prepared by omitting the corresponding primary antibody and adding the secondary IgG isotype antibody.

### Evaluation of ALDH1 and TWIST expression in cancer cell lines using the ARIOL system

Cytospins prepared from all cell lines were triple stained with anti-pancytokeratin, anti-ALDH1 and anti-TWIST antibodies and analyzed with the ARIOL system. Positive and negative controls for each antibody were also prepared.

HepG2 cell line was used as positive control for ALDH1 expression, as proposed by the manufacturer. A differential expression of ALDH1, varying from absent to high was evident among these cells. In order to define the cut-offs between high, low and absent ALDH1 expression, 50 randomly selected microscope vision fields were analyzed and a total of 1.500 cells presenting high, low or no ALDH1 expression (500 cells each) were measured by the ARIOL system. Measurements represent the exposure time required for the detection of ALDH1 fluorescent signal. Using the resulting cut-offs, ALDH1 expression was further evaluated in three representative human breast cancer cell lines: SKBR-3, MCF-7 and MDA.MB.231 (Table 1).

**Table 1:** **Quantification of ALDH1 expression levels in cancer cell lines using the ARIOL system**

ALDH1 expression levels	HepG2		SKBR3		MCF7		MDA.MB.231	
	***Range***	Median ± SE ^a^	***Range***	Median ± SE ^a^	***Range***	Median ± SE ^a^	***Range***	Median ± SE ^a^
**High**	*5 – 25*	15 ± 0.25	*10 – 25*	15 ± 0.23	*20 – 25*	20 ± 0.11	*15 – 25*	20 ± 0.18
**Low**	*30 – 55*	45 ± 0.30	*35 – 55*	45 ± 0.29	*35 – 55*	45 ± 0.29	*30 – 55*	45 ± 0.29
**Negative**	*60 – 90*	70 ± 0.30	*60 – 90*	80 ± 0.39	*60 – 90*	75 ± 0.29	*60 – 100*	80 ± 0.46

^a^SE: standard error.

HepG2 cells were also used as positive control for TWIST expression, since they co-expressed ALDH1 and TWIST. A differential TWIST subcellular localization in nucleus and/or cytoplasm could be observed. In this study, TWIST was characterized as cytoplasmic when localized exclusively in the cytoplasm, and as nuclear when localized in the nucleus, regardless of its co-localization in the cytoplasm. Evaluation of TWIST expression was subsequently performed in SKBR-3, MCF-7 and MDA.MB.231.

### Statistical analysis

Statistical analyses were performed using IBM SPSS Statistics version 20. Chi-square test was used to compare the frequency of CTC phenotypes among early and metastatic breast cancer patients. Mann Whitney test was used to compare the incidence of CTCs with different phenotypes per patient between early and metastatic disease. Spearman’s rho analysis was used to investigate the correlation between specific phenotypes among CTCs. P values were considered statistically significant at the 0.05 level.

## Results

### Sensitivity and specificity of CTC detection

Spiking of breast cancer cell lines into whole blood obtained from healthy donors, revealed that the recovery rates of MCF-7 cells were 53%, 21% and 19% for the dilutions of 1, 10 and 100 cells per ml, respectively. The corresponding values were 27%, 19% and 20% for SKBR-3 and 21%, 21% and 31% for MDA.MB.231 cells.

Spiking of MCF-7 cells into PBMCs showed recovery rates of 80% for the dilution of 1 cell per 1*10^6^ PBMCs and 100% for the dilutions 10 and 100 cells per 1*10^6^ PBMCs.

**Figure 1:**
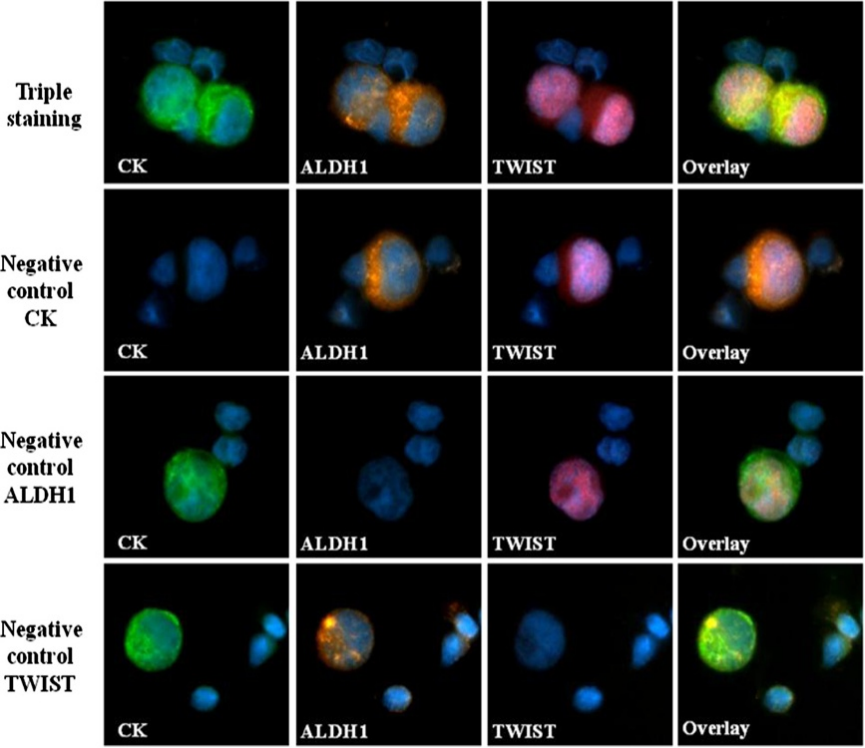
**Control experiments for the specificity of Cytokeratin, ALDH1 and TWIST antibodies in HepG2 cells spiked in PBMCs, ARIOL system.** Triple immunofluorescence was performed in cytospin preparations of HepG2 cells spiked in PBMCs from healthy blood donors, using anti-Cytokeratin (green), anti-ALDH1 (orange) and anti-TWIST (pink) antibodies. Negative controls were prepared for each primary antibody, by omitting the corresponding primary antibody and adding the secondary IgG isotype antibody. Cell nuclei were stained with Dapi (blue), ARIOL system (x400).

No cytokeratin-positive cells could be detected in PBMCs’ cytospins from healthy donors; however, expression of both ALDH1 and TWIST could be identified among PBMCs in all samples analyzed.Evaluation of cytospins from HepG2 cells spiked into PBMCs, prepared as positive and negative controls, showed high specificity for all the antibodies used in the current assay (Figure 1). Spiked HepG2 were included as controls in each separate immunofluorescence experiment performed for patient samples.

### Definition of high and low ALDH1 expression levels and characterization of TWIST sub-cellular localization in cancer cell lines

**Figure 2:**
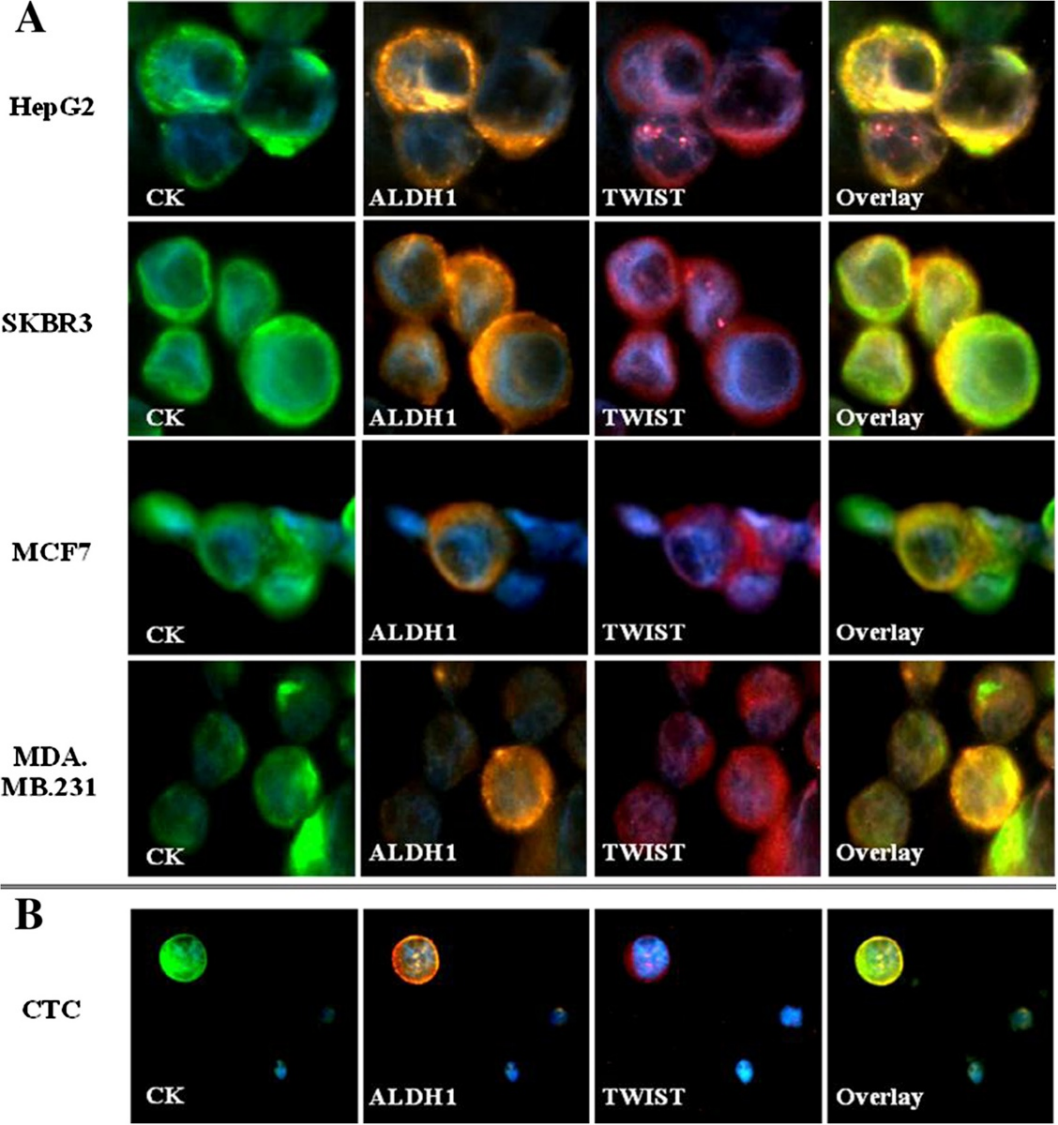
**Co-expression of Cytokeratin, ALDH1 and TWIST in cancer cell lines and a single CTC detected in a breast cancer patient, ARIOL system.** Triple immunofluorescence was performed in cytospin preparations using anti-CK (green), anti-ALDH1 (orange) and anti-TWIST (pink) antibodies. Cell nuclei were stained with Dapi (blue). **A)** HepG2 control cells and three representative breast cancer cell lines, ARIOL system (x400). **B)** A CTC (ALDH1^high^/TWIST^nuc^ phenotype) detected in a metastatic breast cancer patient, ARIOL system (x200).

HepG2 cell line was used as control for the evaluation of ALDH1 expression levels. High ALDH1 expression was evident in the great majority of HepG2 cells; however cells presenting low or absent ALDH1 expression were also observed (Figure 2A, Additional file 1A). Measurements (exposure time) for high ALDH1 expression levels ranged from 5 to 25 (median: 15 ± 0.25), while low ALDH1 expression levels ranged from 30 to 55 (median: 45 ± 0.30). Hence, high ALDH1 expression (ALDH1^high^) was defined at measurements of 25 or lower, whereas low ALDH1 expression (ALDH1^low^) was defined at measurements between 30 to 55. The absence of ALDH1 expression (ALDH1^neg^) was also evaluated by the use of negative controls, at measurements of 60 and higher (range: 60–90, median: 70 ± 0.30). The range of the measurements and the median values with standard error (SE) within the ALDH1^high^, ALDH1^low^ and ALDH^neg^ cell populations are presented in Table 1.

Using the above cut-off points, ALDH1 expression was subsequently evaluated in three human breast cancer cell lines: SKBR-3, MCF-7 and MDA.MB.231, representative of the three breast cancer subtypes: HER2-positive (Human Epidermal Growth Factor Receptor 2), luminal and basal-like, respectively. ALDH1^high^, ALDH1^low^ and ALDH^neg^ cells were detected in all cell lines, with a clear distinction between high, low and absent ALDH1 expression levels (Figure 2A, Additional file 1A). Comparable median values of measurements within the three subpopulations (ALDH1^high^, ALDH1^low^ and ALDH^neg^) were confirmed across HepG2 cells and the three breast cancer cell lines (Table 1).

HepG2 cells were also used as control for the characterization of TWIST expression. TWIST was localized in the nucleus (TWIST^nuc^) in the majority of HepG2 cells; however cells with cytoplasmic TWIST expression (TWIST^cyt^) and cells lacking TWIST expression (TWIST^neg^) were also observed. TWIST^nuc^, TWIST^cyt^ and TWIST^neg^ cells were also detected in all breast cancer cell lines (Figure 2A, Additional file 1B). Co-expression of ALDH1 and TWIST was also confirmed in all cell lines.

### Expression of ALDH1 and TWIST in CTCs of patients with early breast cancer

CTCs were detected in 13 out of 80 (16.3%) patients, with a total of 31 CTCs identified [median No. CTCs/ patient: 1 (range: 1–6)].

#### ALDH1 expression

ALDH1-expressing CTCs were detected in all but one patient; however CTCs with high ALDH1 expression (ALDH1^high^) were observed in 30.8% of patients, whereas 92.3% had detectable CTCs with low or absent ALDH1 (ALDH1^low/neg^) (Table 2). Exclusively ALDH1^high^ and ALDH1^low/neg^ CTCs were identified in 15.4% and 69.2% of patients, respectively. Regarding the distribution of phenotypes at the CTC level, ALDH1^high^ and ALDH1^low/neg^ expression was observed in 38.7% and 61.3% of total CTCs, respectively.

**Table 2:** **Incidence of CTC phenotypes according to differential expression patterns of ALDH1 and TWIST in patients with early and metastatic breast cancer**

***CTC phenotypes***	Patients (%)			Percentage of CTCs per patient (mean; range)			CTCs (%)	
	***Early***	***Metastatic***	***p value***	***Early***	***Metastatic***	***p value***	***Early***	***Metastatic***
**ALDH1 high**	30.8	80.0	*0.009*	23 (0–100)	75 (0–100)	*0.001*	38.7	83.5
**ALDH1 low/neg**	92.3	32.0	*0.006*	77 (0–100)	25 (0–100)	*0.001*	61.3	16.5
**TWIST nuc**	30.8	80.0	*0.009*	29 (0–100)	73 (0–100)	*0.006*	32.3	70.3
**TWIST cyt/neg**	76.9	40.0	*0.040*	71 (0–100)	27 (0–100)	*0.006*	67.7	29.7

Chi-square test (Continuity Correction) and Mann Whitney test were used. Only patients with detectable CTCs were included; early setting: 13 patients and 31 CTCs; metastatic setting: 25 patients and 91 CTCs.

#### TWIST expression

TWIST-expressing CTCs were identified in all but one patient; in 30.8% of patients CTCs with nuclear TWIST localization (TWIST^nuc^) were observed, while 76.9% harvested CTCs with cytoplasmic or absent TWIST expression (TWIST^cyt/neg^) (Table 2). Exclusively TWIST^nuc^ and TWIST^cyt/neg^ CTCs were detected in 23.1% and 69.2% of patients, respectively. Furthermore, the phenotypes TWIST^nuc^ and TWIST^cyt/neg^ were identified in 32.3% and 67.7% of total CTCs, respectively.

#### ALDH1 and TWIST co-expression

Four different phenotypes could be distinguished according to the co-expression of ALDH1 and TWIST at the single CTC level (Table 3). ALDH1^high^/TWIST^nuc^ CTCs were detected in 15.4% of patients, whereas in 61.5% ALDH1^low/neg^/TWIST^cyt/neg^ CTCs were identified. There were no patients presenting exclusively ALDH1^high^/TWIST^nuc^ CTCs, while 53.8% of patients had exclusively ALDH1^low/neg^/TWIST^cyt/neg^ CTCs. Moreover, ALDH1^high^/TWIST^nuc^ and ALDH1^low/neg^/TWIST^cyt/neg^ phenotypes were expressed in 12.9% and 41.9% of total CTCs. The frequency of the two other phenotypes (ALDH1^high^/TWIST^cyt/neg^ and ALDH1^low/neg^/ TWIST^nuc^) among patients and CTCs is also shown in Table 3.

**Table 3:** **Incidence of CTC phenotypes according to the co-expression of ALDH1 and TWIST on single CTCs of patients with early and metastatic breast cancer**

***CTC phenotypes***	Patients (%)			Percentage of CTCs per patient (mean; range)			CTCs (%)	
	***Early***	***Metastatic***	***p value***	***Early***	***Metastatic***	***p value***	***Early***	***Metastatic***
**ALDH1high / TWISTnuc**	15.4	76.0	*0.001*	6 (0–50)	64 (0–100)	*0.000*	12.9	61.5
**ALDH1high / TWISTcyt/neg**	23.1	24.0	*1.000*	17 (0–100)	11 (0–100)	*0.746*	25.8	22.0
**ALDH1low/neg / TWISTnuc**	30.8	12.0	*0.330*	23 (0–100)	8 (0–100)	*0.152*	19.4	8.8
**ALDH1low/neg / TWISTcyt/neg**	61.5	20.0	*0.078*	54 (0–100)	16 (0–100)	*0.026*	41.9	7.7

Chi-square test (Continuity Correction) and Mann Whitney test were used. Only patients with detectable CTCs were included; early setting: 13 patients and 31 CTCs; metastatic setting: 25 patients and 91 CTCs.

A heterogeneous distribution of specific CTC phenotypes in individual patients was observed as shown in Tables 2 and 3, by the differential mean percentages of CTC subpopulations per patient. This variability is further depicted in Table 4 demonstrating the incidence of different CTC phenotypes in index patients with early disease.

**Table 4:** **Distribution of CTC phenotypes according to ALDH1 and TWIST co-expression in index patients with early and metastatic breast cancer**

Patients		ALDH1high/TWISTnuc		ALDH1high/TWISTcyt/neg		ALDH1low/neg/TWISTnuc		ALDH1low/neg/TWISTcyt/neg	
**Early**	***Total CTC No***	***CTC No*** **(%)**		***CTC No*** **(%)**		***CTC No*** **(%)**		***CTC No*** **(%)**	
**1**	*3*	*0*	(0)	*0*	(0)	*0*	(0)	*3*	(100)
**2**	*5*	*0*	(0)	*0*	(0)	*0*	(0)	*5*	(100)
**3**	*3*	*1*	(33.3)	*0*	(0)	*2*	(66.7)	*0*	(0)
**4**	*6*	*3*	(50)	*1*	(16.7)	*2*	(33.3)	*0*	(0)
**5**	*6*	*0*	(0)	*6*	(100)	*0*	(0)	*0*	(0)
**Metastatic**									
**1**	*2*	*2*	(100)	*0*	(0)	*0*	(0)	*0*	(0)
**2**	*11*	*10*	(90.9)	*1*	(9.1)	*0*	(0)	*0*	(0)
**3**	*2*	*1*	(50)	*0*	(0)	*1*	(50)	*0*	(0)
**4**	*3*	*1*	(20)	*2*	(80)	*0*	(0)	*0*	(0)
**5**	*21*	*5*	(23.8)	*14*	(66.7)	*0*	(0)	*2*	(9.5)
**6**	*2*	*0*	(0)	*0*	(0)	*0*	(0)	*2*	(100)
**7**	*11*	*3*	(27.2)	*2*	(18.2)	*6*	(54.5)	*0*	(0)

### Expression of ALDH1 and TWIST in CTCs of patients with metastatic breast cancer

The presence of CTCs was documented in 25 out of 50 (50%) patients, with a total of 91 CTCs detected [median No. CTCs/ patient: 2 (range: 1–21)].

#### ALDH1 expression

ALDH1-expressing CTCs were evident in all patients; however, ALDH1^high^ CTCs were detected in 80% of patients (p = 0.009, compared to early disease), whereas ALDH1^low/neg^ CTCs were observed in 32% (p = 0.006) (Table 2). Exclusively ALDH1^high^ and ALDH1^low/neg^ CTCs were detected in 68% and 20% of patients (p = 0.006 and p = 0.009, respectively, compared to early patients). Moreover, ALDH1^high^ and ALDH1^low/neg^ was identified in 83.5% and 16.5% of total CTCs, respectively.

#### TWIST expression

TWIST-expressing CTCs were also detected in all patients; however TWIST^nuc^ CTCs were identified in 80% of patients, while TWIST^cyt/neg^ were observed in 40% (p = 0.009 and p = 0.040, compared to early disease) (Table 2). Exclusively TWIST^nuc^ and TWIST^cyt/neg^ CTCs were detected in 64% (p = 0.040) and 20% (p = 0.009) of patients. Furthermore, the phenotypes TWIST^nuc^ and TWIST^cyt/neg^ were observed in 70.3% and 29.7% of total CTCs, respectively.

#### ALDH1 and TWIST co-expression

Evaluation of ALDH1 and TWIST co-expression on single CTCs showed that 76% of patients harvested ALDH1^high^/TWIST^nuc^ CTCs (p = 0.001, compared to early patients), whereas 20% had detectable ALDH1^low/neg^/TWIST^cyt/neg^ CTCs (p = 0.078) (Table 3). Exclusively ALDH1^high^/TWIST^nuc^ and ALDH1^low/neg^/TWIST^cyt/neg^ CTCs were detected in 56% (p = 0.002) and 16% (p = 0.078) of patients, respectively. In the CTC level, the phenotypes ALDH1^high^/TWIST^nuc^ and ALDH1^low/neg^/TWIST^cyt/neg^ were confirmed in 61.5% and 7.7% of total CTCs, respectively. The incidence of ALDH1^high^/TWIST^cyt/neg^ and ALDH1^low/neg^/TWIST^nuc^ CTCs was similar to early disease (Table 3). As shown for early disease, distinct CTC phenotypes could be observed in individual metastatic patients (Tables 3 and 4). An ALDH1^high^/TWIST^nuc^ CTC is depicted in Figure 2B.

Finally, a positive correlation between ALDH1^high^ and TWIST^nuc^ expression was confirmed on CTCs of metastatic patients (p = 0.001, Spearman’s rho analysis), whereas ALDH1^low/neg^ was associated with TWIST^cyt/neg^ (p = 0.001).

## Discussion

CTCs are considered to be the active source of metastatic spread; however only a few of these cells are capable of forming metastatic deposits in distant organs. Indeed, although the presence of CTCs in patients with breast cancer has been associated with poor prognosis [2, 4], many patients do not relapse even when CTCs are detected in their blood. Thus, besides detection, further phenotypic characterization of these cells might provide additional information for their metastatic potential.

Metastasis is a complex multistep cascade of events and cancer cells need to be highly equipped in order to fulfill the metastatic process. CSCs are suggested to have the ability to self-renew and regenerate the tumor [8]. Moreover, EMT has been linked to cancer progression and acquisition of stem cell-like properties [32]. Thus, CTCs co-expressing stem cell and EMT markers could be actively involved in tumor progression. We have reported that the stemness markers CD44/CD24 and ALDH1 are expressed in CTCs of patients with metastatic breast cancer [14]. Moreover, we have recently shown that the EMT markers TWIST and Vimentin were frequently expressed on CTCs of patients with early and metastatic breast cancer [22]. In this study, we developed a new methodology to investigate the expression pattern of ALDH1 and TWIST on CTCs of breast cancer patients and to evaluate their co-expression at the single CTC level.

The expression of ALDH1 in primary tumors has been associated with poor patient outcome in several cancers, including breast cancer [10, 12, 33]. Moreover, differential ALDH1 expression levels have been demonstrated and a positive correlation has been suggested between high ALDH1 and worse clinical outcome [34–36]. High ALDH1 protein expression has also been associated with high ALDH enzymatic activity, a putative marker for CSCs [37]. Accordingly, in the present immunofluorescence assay, a quantitative analysis of ALDH1 expression levels by the use of the ARIOL system software was employed [22].

With the provided quantification method, a clear distinction between high and low ALDH1 expression was demonstrated in HepG2 control cell line. The evaluation of ALDH1 expression in three breast cancer cell lines representative of HER2-positive, luminal and basal-like subtypes, further confirmed the presence of ALDH1^high^, ALDH1^low^ and ALDH^neg^ cells within each cell line. The comparable range and median expression values of each cell subpopulation among all cell lines verified the objectivity of ALDH1 quantification irrespectively of the specific breast cancer subtype and allowed its application on patient samples.

Interestingly, although ALDH1-expressing CTCs were identified in almost all CTC-positive patients, the pattern of ALDH1 expression differed among CTCs in both clinical settings. Moreover, ALDH1^high^ CTCs were more frequently observed in metastatic patients, whereas ALDH1^low/neg^ CTCs were mainly detected in patients with early disease. This observation suggests that ALDH1^high^ CTCs predominate during disease progression and leads to the assumption that CTCs bearing stemness characteristics may have an active role in the metastatic process. We have previously reported a lower frequency of ALDH1^high^ CTCs in patients with metastatic breast cancer, which could be explained by the lower number of patients included in that study, as well as by the different methodologies used for the titration of ALDH1 expression [14].

TWIST is a transcription factor with a pivotal role in EMT induction, both in normal and cancer cells [38]. The expression of TWIST in breast tumors has been correlated to increased metastatic potential and poor survival [19]. In the present study, we further analyzed the subcellular localization of TWIST on CTCs, since efficient nuclear localization is essential for a protein to operate as an activator and/or repressor of transcription of target genes [39]. Furthermore, Yuen et al. showed that nuclear TWIST localization predicted the metastatic potential of prostate tumors [40], whereas in esophageal squamous cell carcinoma, it was associated with lymph node metastasis [41]. The data presented in the current study are in agreement with our previously reported results showing that TWIST is expressed in the majority of CTCs derived from patients with breast cancer [22]. Here we further show that CTCs present a differential TWIST subcellular localization pattern. In addition, we demonstrate that TWIST^nuc^ CTCs were more frequently detected in metastatic patients, while in early disease TWIST^cyt/neg^ CTCs were mainly observed. This observation suggests that TWIST localization may be related with functional cellular properties during the different stages of the disease. It could be hypothesized that TWIST^nuc^ CTCs are undergoing EMT and selected during disease progression. In accordance, a recent study showed that CTCs of breast cancer patients exhibit dynamic changes in epithelial and mesenchymal composition and that the presence of CTCs in EMT state was associated with disease progression [42].

Previous studies have also reported the expression of ALDH1 and TWIST on CTCs of early and metastatic breast cancer patients [27, 43], though at a lower frequency. This could be attributed to methodological differences, since the *AdnaTest* used in these studies analyzes mRNA expression in CTC-positive blood samples, whereas in the current assay protein expression on single CTCs is evaluated.

Using the present assay, four different CTC phenotypes were identified according to the simultaneous evaluation of both markers. An interesting finding was the considerable inter- and intra-patient heterogeneity regarding the frequency of distinct CTC subpopulations either in the early or the metastatic disease setting. Moreover, a differential distribution of phenotypes was evident comparing the two groups of patients; ALDH1^high^/TWIST^nuc^ CTCs were more prominent among metastatic patients, whereas the ALDH1^low/neg^/TWIST^cyt/neg^ phenotype predominated in patients with early disease. The finding that ALDH1^high^ and TWIST^nuc^ phenotypes were mainly co-expressed in the same CTC, as well as their positive correlation shown in metastatic disease, further supports the hypothesis of a link between stemness and EMT characteristics on cancer cells. [44, 45]. This is also in agreement with recent studies showing that overexpression of TWIST induces ALDH1 expression in cell lines [46, 47].

In the current study, CTCs bearing high ALDH1 expression, along with nuclear TWIST localization, are not proven to be cancer stem cells undergoing EMT. Further experiments with functional assays would be required to validate their stemness and EMT properties. Nevertheless, this is beyond the scope of the current report which aimed in the evaluation of previously suggested stemness and EMT markers on single CTCs. The higher prevalence of these markers in metastatic breast cancer patients suggests that they could possibly distinguish a subpopulation of CTCs with aggressive biological properties. Therefore, phenotypic characterization of CTCs according to the expression of ALDH1 and TWIST merits further evaluation in a larger cohort of patients, in order to investigate the clinical significance of the above findings.

## Conclusions

The current study provides a new methodology for the evaluation of ALDH1 and TWIST co-expression on single CTCs of patients with breast cancer. Using this assay, distinct CTC phenotypes, according to ALDH1 expression levels and TWIST subcellular localization, were designated in patients with early and metastatic breast cancer. The higher incidence of CTCs bearing putative stem cell and EMT traits in metastatic disease, suggests that these characteristics may prevail on CTCs during disease progression. A correlation between stemness and EMT features was further confirmed on single CTCs.

## Electronic supplementary material

Additional file 1: **Expression of ALDH1 and TWIST in cancer cell lines, ARIOL system.** Single immunofluorescence was performed in cytospin preparations from HepG2 control cells and three breast cancer cell lines, ARIOL system (x400). The different phenotypes according to the expression pattern of ALDH1 and TWIST are shown indicatively in MCF7 cells. A) ALDH1^high^, ALDH1^low^ and ALDH1^neg^ cells were observed within all cell lines, by staining with anti-ALDH1 antibody (orange). B) TWIST^nuc^, TWIST^cyt^ and TWIST^neg^ cells were detected within each cell line, using an anti-TWIST antibody (pink). Cell nuclei were stained with Dapi (blue). (PDF 62 KB)
