# Supplementary figures and images for: Co-expression of putative stemness and epithelial-to-mesenchymal transition markers on single circulating tumour cells from patients with early and metastatic breast cancer
Source: BMC Cancer. 2014 Sep 3;14:651. doi: 10.1186/1471-2407-14-651 (PMC4161777; doi:10.1186/1471-2407-14-651)

**A**

*ALDH1*

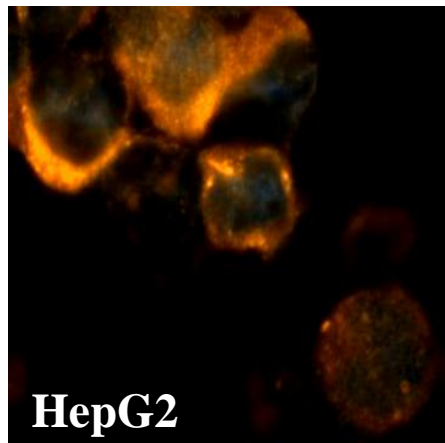

HepG2

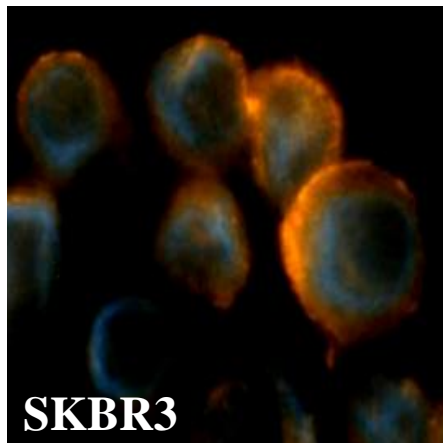

SKBR3

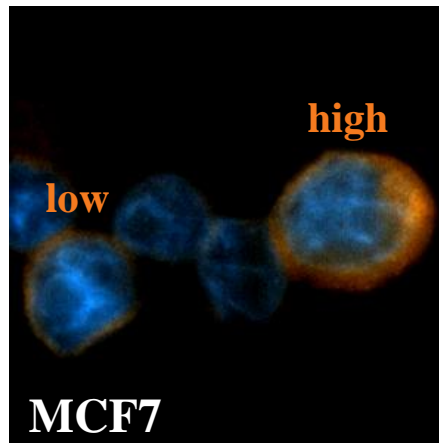

MCF7

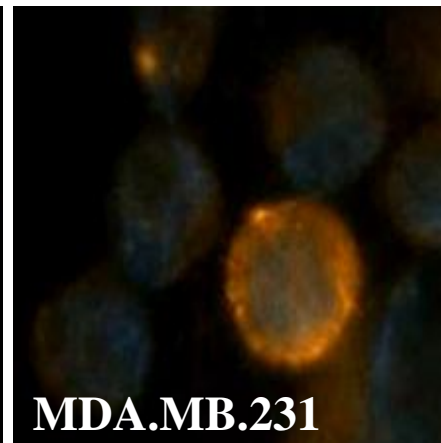

MDA.MB.231

**B**

*TWIST*

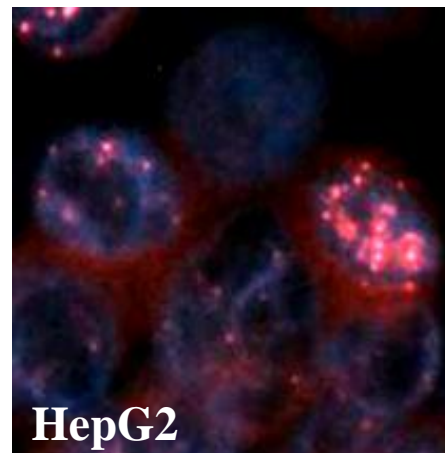

HepG2

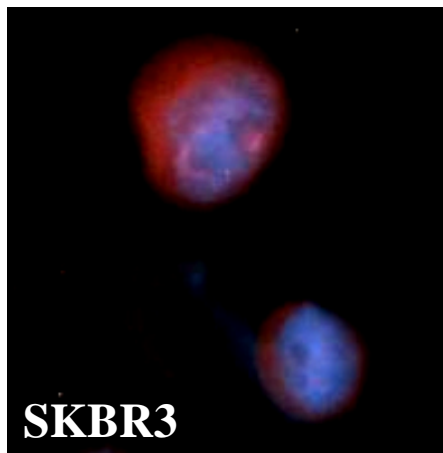

SKBR3

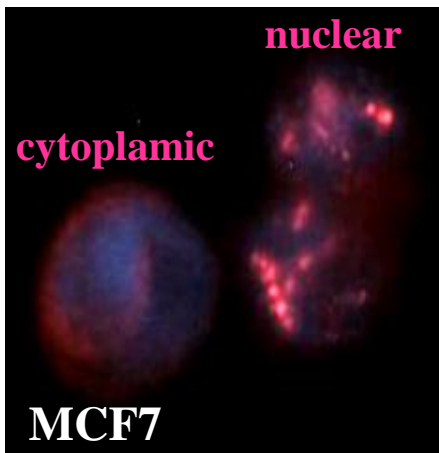

MCF7

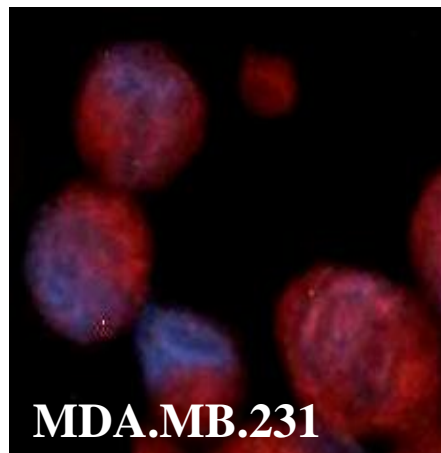

MDA.MB.231

Supplement: Supplementary file 1 — Additional file 1: Expression of ALDH1 and TWIST in cancer cell lines, ARIOL system. Single immunofluorescence was performed in cytospin preparations from HepG2 control cells and three breast cancer cell lines, ARIOL system (x400). The different phenotypes according to the expression pattern of ALDH1 and TWIST are shown indicatively in MCF7 cells. A) ALDH1high, ALDH1low and ALDH1neg cells were observed within all cell lines, by staining with anti-ALDH1 antibody (orange). B) TWISTnuc, TWISTcyt and TWISTneg cells were detected within each cell line, using an anti-TWIST antibody (pink). Cell nuclei were stained with Dapi (blue). (PDF 62 KB) [file 12885_2013_4826_MOESM1_ESM.pdf]
